# The Intertwined Life Cycles of Enterovirus and Autophagy

**DOI:** 10.1080/21505594.2018.1551010

**Published:** 2018-12-03

**Authors:** Yasir Mohamud, Honglin Luo

**Affiliations:** Center for Heart Lung Innovation, St. Paul’ s Hospital and Department of Pathology and Laboratory Medicine, University of British Columbia, Vancouver, BC, Canada

**Keywords:** Enterovirus, autophagy, autophagosome, amphisome, lysosome, non-lytic release, virophagy, immune evasion, secretory autophagy

## Abstract

Enteroviruses (EVs) are the most common human pathogens worldwide. Recent international outbreaks in North America and South East Asia have emphasized the need for more effective anti-viral therapies. As obligate parasites, EVs rely on the host cellular machinery for effective viral propagation. Accumulating evidence has indicated that EVs subvert and disrupt the cellular autophagy pathway to facilitate productive infection, and consequently leading to host pathogenesis. Given that defective autophagy is a common factor in various human diseases, including neurodegeneration, cardiomyopathy, and metabolic disorders, a clear understanding of the relationship between EV infection and autophagy is warranted. In this review, we highlight recent advances in understanding the molecular mechanisms by which EVs exploit the autophagy pathway during different steps of viral life cycle, from entry, replication, and maturation to release. We also provide an overview of recent progress in EV subversion of the autophagy for immune evasion.

## Introduction

Enteroviruses (EVs), of the *Picornaviridae* family, are a genus of pathogenic viruses that cause diverse human disorders. The major members of the EV genus include poliovirus (PV), echovirus, coxsackievirus, enterovirus, and rhinovirus [1]. These medically important viruses pose a significant health burden as evidenced by recent global epidemics across Asia and North America [2,3]. EVs are associated with broad human pathologies, ranging from the common cold to neurological disorder (i.e., encephalitis, aseptic meningitis, and flaccid paralysis) [4], cardiovascular damage (i.e., viral myocarditis and dilated cardiomyopathy) [5], and metabolic disease (i.e., type 1 diabetes) [6]. With the exception of the successful PV vaccination beginning in the 1950s, non-polio EVs continue to be a global health issue.

EVs are a family of small, non-enveloped viruses containing a positive, single-stranded RNA genome of ~7.5 kb that encodes a single open reading frame flanked by 5ʹ and 3ʹ untranslated regions (UTRs) [1]. The life cycle of an EV begins with viral attachment to one or multiple designated cellular receptors. Following internalization, viral RNA is released into the cytoplasm, where it serves as a template for the translation of the viral polyprotein and for the replication of the viral genome. The 5ʹUTR of EV genome contains an internal ribosome entry site (IRES) for viral translation initiation, which allows EVs to bypass the shutoff of cap-dependent protein translation that occurs during viral infection [7]. Viral proteins are initially synthesized as a single polyprotein, which is subsequently processed into structural proteins (VP1, VP2, VP3, and VP4), nonstructural proteins (2A, 2B, 2C, 3A, 3B, 3C, and 3D), and cleavage intermediates by virus-encoded proteases 2A and 3C (or 3CD, the precursor). The viral RNA-dependent RNA polymerase 3D then uses protein 3B (also known as viral protein genome-linked (VPg) that is covalently linked to the 5ʹ-end of viral genome) as a primer for both negative- and positive-strand RNA synthesis. Viral replication takes place on virus-modified intracellular membranous structures that not only serve as physical scaffolds but also provide favorable lipid compositions for viral RNA assembly and replication [8,9]. The viral proteins 2B, 2C (or their precursor 2BC), and 3A (or its precursor 3AB) regulate cellular permeability and vesicular transport, and constitute essential components of the viral replication complexes [10]. Finally, the newly synthesized RNA genome is packaged into a viral capsid of ~30 nm in size to generate the nascent infectious viral particle. The life cycle is completed when viral progeny are released from the infected cells following cell lysis or non-cytolytically prior to cell rupture through extracellular microvesicles [11–14].

Autophagy is a cellular process that targets cytoplasmic proteins and/or organelles to the lysosomes for degradation [15,16]. Over the past decade, extensive research has been conducted to explore how EVs evolve to harness the cellular autophagy pathway to facilitate the successful completion of their life cycle. The current review will focus on the recent progress in understanding the interplay between EV infection and the autophagy pathway. Specifically, we will provide an overview of how EVs hijack components of this cellular process at the different steps of viral life cycle to promote viral growth and for immune evasion.

## Autophagy

Autophagy is an evolutionarily conserved intracellular degradation pathway that plays a key role in maintaining cellular homeostasis under both normal and stress conditions [15,16]. It exists in three forms, i.e., macroautophagy, chaperone-mediated autophagy, and microautophagy [17]. In this review, we focus on macroautophagy as all current literature about EV-autophagy interaction has been on this type of autophagy. The process of macroautophagy (hereafter referred to as autophagy) begins with the formation of phagophore (also known as the isolation membrane), a cup-shaped double-membraned structure that sequesters cellular components. Although the origin(s) of the phagophore membrane remain not fully elucidated, multiple sources, including the endoplasmic reticulum (ER) [18], mitochondria [19], ER-mitochondria contact sites/mitochondria-associated membranes [20], ER-Golgi intermediate compartments [21], endosomes, and plasma membrane [22], have been proposed. Following the closure of the phagophore, a double-membraned vesicle, so-called autophagosome, is generated. The autophagosome or amphisome (the latter is formed by the merge of an autophagosome with an endosome) then fuses with a lysosome to form autolysosome and the cargo enwrapped including the inner membrane of the autophagosome is degraded by hydrolysis [16].

In mammalian cells, the molecular processes of autophagy are controlled by a set of more than 30 ‘autophagy-related’ (ATG) genes. Proteins encoded by these genes participate in the initiation and formation of autophagosomes through the action of several protein complexes and molecules [23,24]. Upon autophagy stimulation, the uncoordinated (UNC)-51-like kinase (ULK) complex, consisting of ULK1/2, ATG13, RB1CC1 (RB1 inducible coiled-coil 1)/FIP200 (focal adhesion kinase family interacting protein of 200 kDa), and ATG101, translocates to the autophagy initiation sites and facilitates the recruitment of the class III phosphatidylinositol-3-kinase (PI3K) complex. The PI3K complex is composed of PI3KC3 (phosphatidylinositol 3-kinase catalytic subunit type 3)/VPS34 (vacuolar protein sorting 34), PIK3R4 (phosphoinositide 3-kinase regulatory subunit 4)/VPS15, AMBRA1 (activating molecule in Beclin-1-regulated autophagy protein 1), ATG14, and Beclin-1, which promote autophagosome formation by providing PtdIns(3)P (PI3P) to the phagophores. The PI3P then recruits the downstream ATG2-WIPI (WD-repeat protein interacting with phosphoinositides) complex, comprising of transmembrane protein ATG9, WIPI, and ATG2, to the phagophore, which in turn facilitates the recruitment of ATG5-ATG12-ATG16L1 complex and covalent association of LC3 (microtubule-associated protein light chain 3) to phosphatidylethanolamine (PE) on the nascent autophagosome membrane, allowing for autophagosome maturation.

The fusion between autophagosomes and lysosomes is regulated by multiple proteins involved in the intracellular membrane trafficking, including the Rab GTPases, the soluble N-ethylmaleimide-sensitive factor (NSF)-activating protein receptor (SNARE) proteins, and the membrane-tethering proteins [25]. Upon autophagy induction, the SNARE protein syntaxin 17 (STX17), is recruited to autophagosomes, where it binds to another autophagosomal SNARE protein, the synaptosomal-associated protein 29 (SNAP29) to form a binary SNARE protein complex (STX17-SNAP29) [26,27]. ATG14 is then recruited to the binary SNARE complex to facilitate the generation of a ternary SNARE complex with the lysosome/endosome-localized SNARE protein, vesicle-associated membrane protein 8 (VAMP8), driving autophagosome-lysosome fusion [28]. Beyond the SNARE proteins, several membrane-tethering proteins are also involved in the fusion step of autophagy. For example, the pleckstrin homology domain containing protein family member 1 (PLEKHM1) protein was reported to interact with Rab7, homotypic fusion and protein sorting (HOPS), and LC3 to promote the formation of SNARE complexes during autophagosome fusion [29,30]. For a more in-depth review of autophagic fusion requirements, please refer to Corona *et al* [31].

Autophagy can be induced by various stimuli and stressors, including nutrient starvation, metabolic and oxidative stress (reactive oxygen and nitrogen species), ER stress, hypoxia, infectious agents, and the presence of damaged organelle, through either a cargo-selective or a cargo–non-selective pathway [32,33]. Under nutrient-stress or normal conditions, autophagy initiates a non-selective degradation of cytoplasmic contents for energy preservation. However, autophagy can also facilitate selective clearance of cytoplasmic materials in response to specific cellular and/or environmental stimuli (termed selective or precision autophagy), such as protein aggregates, damaged mitochondria, and invading pathogens in a process termed the aggrephagy, mitophagy, and xenophagy, respectively [16]. Cargo specificity is mediated by a family of multivalent autophagy receptors, which harbor ubiquitin-association domains (e.g., UBA (ubiquitin-associated) or UBZ (ubiquitin-binding zinc finger)) for substrate recognition, and LC3-interacting regions (LIRs) for binding to the autophagosome-localized LC3, thereby bridging ubiquitinated substrates to the autophagosomes for degradation [34,35]. To date, a number of autophagy receptors have been identified, including sequestosome 1 (SQSTM1)/p62, neighbor of BRCA1 (NBR1), optineurin, calcium binding and coiled-coil domain-containing protein 2 (CALCOCO2)/nuclear dot 10 protein 52 (NDP52), and CALCOCO3/Tax1-binding protein1 (TAX1BP1).

**Figure 1.**
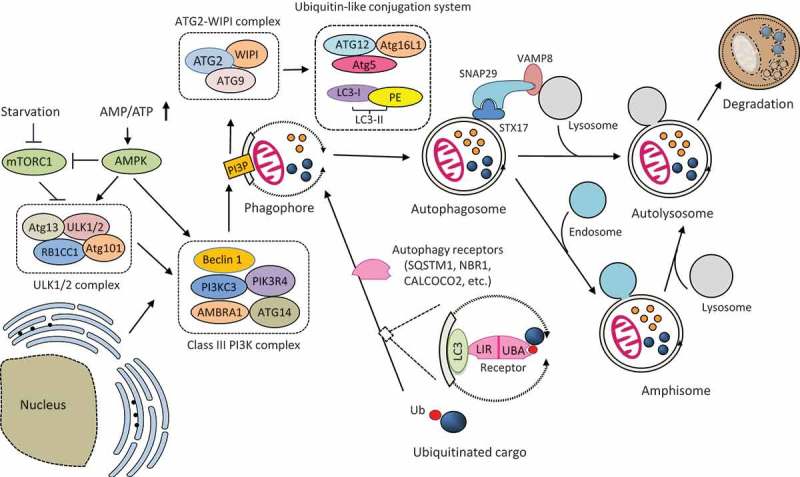
Schematic illustration of the molecular mechanism of autophagy. Autophagosome formation is initiated by the nutrient-sensing kinases (i.e., mTORC1 and AMPK) and through the function of three protein complexes (i.e., ULK1/2 complex, class III PI3K complex, and ATG2-WIPI complex) and two ubiquitin-like conjugation systems (i.e., ATG12-ATG5-ATG16L1 and LC3-PE). Once formed, the autophagosome then fuses with the lysosome to produce an autolysosome. Autophagosome can also merge with the endosome to form an amphisome, which then fuse with a lysosome for cargo degradation. The fusion between an autophagosome and a lysosome is regulated by multiple proteins, particularly the (STX17-SNAP29-VAMP8) SNARE complex. Selective cargo degradation is mediated by autophagy receptors, such as SQSTM1, NBR1, and CALCOCO2, which harbor a UBA domain for substrate recognition, and a LIR for binding to autophagosome-anchored LC3-II, thereby bridging the substrate to the autophagosome for degradation. mTORC1, mechanistic target of rapamycin complex 1; AMPK, AMP activated protein kinase; ATG, autophagy-related; ULK, uncoordinated (UNC)-51-like kinase; RB1CC1, RB1 inducible coiled-coil 1; PI3KC3, phosphatidylinositol 3-kinase catalytic subunit type 3; PIK3R4, phosphoinositide 3-kinase regulatory subunit 4; AMBRA1, activating molecule in Beclin-1-regulated autophagy protein 1; PI3P, PtdIns(3)P; WIPI, WD-repeat protein interacting with phosphoinositides; LC3, microtubule-associated protein light chain 3; PE, phosphatidylethanolamine; SQSTM1, sequestosome 1, NBR1, neighbor of BRCA1; CALCOCO2, calcium binding and coiled-coil domain-containing protein 2; SNAP29, synaptosomal-associated protein 29; STX17, syntaxin 17; VAMP8, vesicle-associated membrane protein 8; LIR, LC3-interacting region; UBA, ubiquitin-associated; Ub, ubiquitin.

The molecular mechanism of autophagy is illustrated in Figure 1.

**Figure 2.**
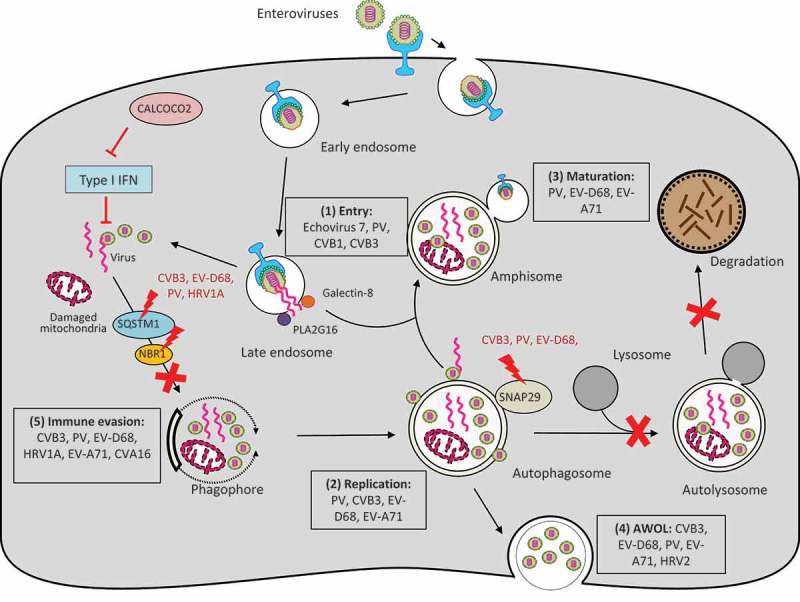
Interplay between enteroviruses and the host autophagy pathway. Major members of enteroviruses have been shown to subvert the host autophagy machinery at different phases of viral life cycle to enhance viral growth and for immune escape. (1) Viral entry. Autophagy core proteins benefit echovirus 7 entry, likely through regulation of the endocytic pathway. During PV, CVB1, and CVB3 infection, PLA2G16 is recruited to the fragmented endosome to antagonize the function of galectin-8 in initiating autophagic degradation of viral RNA genome. (2) Viral replication. Autophagosome-lysosome fusion is blocked during CVB3, PV, and EV-D68 infection through viral protease 3C-induced cleavage of SNAP29. Accumulation of autophagosomes appears to favor viral replication by offering replication membranes for PV, CVB3, EV-D68, and EV-A71. (3) Viral maturation. Formation of acidified amphisomes is required for EV (e.g., PV, EV-D68, EV-A71) maturation to generate infectious viruses. (4) Viral release through AWOL. EVs (e.g., PV, EV-D68, EV-A71, CVB3, and HRV2) utilize autophagosomes as envelopes for non-lytic viral exit. (4) Immune evasion. Autophagy receptor SQSTM1 is cleaved by viral protease 2A during CVB3, PV, EV-D68, and HRV1A infection (cleavage of NBR1 is also observed during CVB3 infection) to counteract virophagy-mediated clearance of viral particles/components. CALCOCO2 exhibits a pro-viral function toward CVB3 via inhibiting type I IFN signaling. Enhanced autophagy during EV-A71 and CVA16 infection was shown to benefit viruses by suppressing TLR7-mediated type I IFN signaling. PV, poliovirus; CVB, coxsackievirus B; CVA, coxsackievirus A; EV, enterovirus; HRV, human rhinovirus; SNAP29, synaptosomal-associated protein 29; SQSTM1, sequestosome 1, NBR1, neighbor of BRCA1; CALCOCO2, calcium binding and coiled-coil domain-containing protein 2; AWOL, autophagosome-mediated exit without lysis; IFN, interferon.

Autophagy is generally considered cytoprotective at both baseline and under various forms of stresses by providing energy and degrading damaged proteins/organelles. In addition, autophagy also plays a critical role in host defense [36,37]. As part of the cell autonomous innate immunity, autophagy functions to defend individual cells from invading pathogens such as bacteria, fungi, parasites, and viruses. At the same time, autophagy initiates signals within infected cells to induce systemic immune responses [36,37]. In the context of virus infection, autophagy is classically regarded as an anti-viral mechanism that selectively degrades viral particles or viral components inside lysosomes in a process referred to as virophagy [16]. However, many viruses, including EVs, have evolved to subvert this pathway for pro-viral purposes [38,39] (summarized in Figure 2).

## Endocytic function of autophagy and EV entry

The EVs initiate infections by attachment to host receptors, resulting in receptor-mediated endocytosis, and then undergo uncoating for delivery of the viral genome into the cytoplasm of a target cell. The cellular tropism observed within EVs relies largely on specific receptors available on the target cells. For example, PV receptor (PVR, also known as CD155) [40], intercellular adhesion molecule 1 (ICAM-1) [41], coxsackievirus adenovirus receptor (CAR) [42] and co-receptor decay accelerating factor (DAF, also known as CD55) [43], have been identified as the receptors for PV, major groups of rhinoviruses, and type B coxsackieviruses (CVB), respectively. Despite the discoveries of majority of EV receptors, including the recently identified EV-D68 and coxsackievirus A (CVA)10 receptors [44,45], the precise mechanism of EV entry and the exact site of viral genome penetration and uncoating remain poorly understood. Clathrin-mediated endocytosis is the best-studied, receptor-mediated endocytic pathway for EV internalization. In addition, caveolin-dependent and clathrin/caveolin-independent (macropinocytosis is an example of the latter) endocytic mechanisms are also reported [46].

Echovirus 7 enters polarized intestinal epithelial cells through a clathrin-mediated endocytic mechanism and then proceeds to early and late endosomes for viral genome release [47]. This process was shown to be independent of endosomal acidification, but require the function of Rab7 in membrane trafficking [47]. Rab7 is a small GTPase critical for both endocytosis and autophagy by controlling endo-lysosomal trafficking and autophagosome fusion with lysosomes, respectively [48]. Therefore, it is unclear whether silencing of Rab7 in the above study, impairs the entry of echovirus 7 either through disruption of late endosome maturation, autophagosome fusion with lysosomes, or both. In addition to Rab7, several other core components of autophagy were also discovered to be involved in the regulation of the endocytic pathway. For instance, Beclin 1 was shown to control early endosome formation as well as late endosome trafficking and maturation through the UVRAG (UV radiation resistance-associated gene)-PI3KC3/VPS34 complex [49]. It was also reported that conjugation of the ubiquitin-like molecule ATG12 to ATG3 (an E2-like enzyme that catalyzes LC3 lipidation) mediates endo-lysosomal trafficking and late endosome function beyond autophagy [50]. Furthermore, ATG5 and ATG16L1 have recently been linked to endosomal acidification and exosome biogenesis independent of the canonical autophagy machinery [51]. Given the demonstrated significance of autophagy core proteins in regulating the endocytic pathway and the recognized cross-talk between the endosomal and autophagic pathways via the formation of amphisomes, Kim *et al* [52]. further investigated the possible involvement of other autophagy-related proteins in echovirus 7 entry. They found that knockdown of Beclin-1, ATG12, ATG14, ATG16L1, or LC3 prevents internalization of echovirus 7 but not CVB3 at a step after viral attachment to its receptor but prior to uncoating [52]. Although this process appears to be independent of autophagosome and amphisome formation, a possible role in overall membrane trafficking has not been excluded [52]. The mechanism associated with this virus-specific requirement for autophagy proteins in EV entry is not clear; it may be related to differential endocytotic mechanism (clathrin-dependent versus caveolin-dependent for echovirus 7 and CVB3 entry, respectively) in polarized cells [53].

Another example linking autophagy to EV entry is the recent finding that during early viral infection (PV, CVB1, and CVB3) host factors are recruited to the ruptured endosomes to escape the autophagy-mediated degradation of viral RNA [54]. Using haploid genetic screens, Staring *et al* [54]. identified PLA2G16, a small phospholipase, to be a key regulator for viral genome delivery into the cytoplasm. Further screen for gene mutations that can rescue viral infection in PLA2G16-deficient cells demonstrated an inhibitory role for galectin-8 in EV infection by promoting autophagy degradation of viral RNA genome [54]. A model is proposed to explain the competitive determination of the fate of viral RNA at the interface of endosomes: after viral endocytosis, endosome fragmentation occurs, leading to the exposure of β-galactosides on the luminal side of the endosomes to galectin-8 and consequently initiating selective autophagy for viral clearance; to compete with this process, PLA2G16 is recruited to the permeated endosomes, allowing for effective viral RNA release into the cytoplasm for translation [54].

## Induction of autophagy and EV replication

Similar to other positive-strand RNA viruses, replication of EVs occurs on distinct intracellular membranes, which are modified both architecturally and chemically by EVs to assemble viral replication complexes (also defined as viral replication organelles [8,9,55]. Studies have revealed that EVs modulate host lipid metabolism/distribution and utilize membrane complexes enriched in phosphatidylinositol 4-phosphate (PI4P) and cholesterol for replication [55–57]. Despite notable progress in understanding cellular membrane remodeling and its significance in EV infection, the contribution of autophagy in such event remains largely undefined.

It has been well documented that EV infection induces the formation of autophagy-associated membrane scaffolds, favoring viral RNA synthesis. Almost all major EVs, including PV [58], CVB3 [59,60], CVB4 [61], CVA16 [62], EV-A71 [63,64], EV-D68 [65], and human rhinovirus 2 (HRV2) [66], have been shown to facilitate the production of autophagosomes, as demonstrated by LC3 lipidation and puncta formation, as well as detection of intracellular double-membraned vesicles. Disruption of autophagy using chemical inhibitors (such as 3-methyladenine, a class III PI3K inhibitor) or through deletion of critical genes involved in autophagosome formation/maturation (such as ATG5, ATG7, Beclin 1, PI3KC3/VPS34, ATG14, and UVRAG) inhibits viral replication both *in vitro* and *in vivo* [60,65,67–69]. The mechanism underlying the pro-viral function of autophagy is still unclear. Electron microscopy analysis of CVB3-infected acinar cells revealed that the highly organized lattice structures (likely viral replication organelles) observed in normal mice are barely detected in ATG5-deficient mice [59,67]. In addition, research utilizing an antibody against double-stranded RNA (dsRNA, a replication intermediate of viral RNA used as a marker for viral replication complexes) showed that dsRNA colocalizes with LC3-positive puncta (as a marker for autophagosomes and autophagy-independent LC3 accumulation) during late PV infection [70], in line with the 3D ultrastructural results that PV induces early convoluted single-membraned structures and late autophagosome-like double-membraned vesicles [71]. These findings, together with recent evidence that PI4P lipids accumulate on autophagosomes upon autophagy stress to promote autophagosome-lysosome fusion [72], support the premise that autophagosomes serve as sites, at least during late phase of maximal viral replication, for viral RNA replication. It is important to note that previous studies on the role of autophagy in EV replication have been conducted predominantly through deletion of individual autophagy-related genes. Some of the ATG proteins are known to have unconventional functions beyond autophagy [73,74]. Thus, further investigation is required to determine whether the pro-viral role of these ATG proteins is truly or exclusively via the autophagy pathway. Indeed, both autophagy-dependent and – independent function of LC3 has been described to benefit CVB3 replication *in vivo* [75]. Moreover, knockdown of ATG13 and RB1CC1/FIP200, components of the ULK1/2 complex, has been found to enhance CVB3, EV-A71, and CVA21 replication; this effect seems to be unrelated to autophagy function as depletion of genes encoded for other ULK1/2 components (such as ULK1, ULK2, and ATG101) does not affect viral replication [76].

Although the precise mechanism by which EVs induce autophagy remains to be elucidated, several studies have examined the upstream pathways that can potentially be co-opted to initiate autophagy. Canonical autophagy receives signaling input through nutrient-sensing kinases such as AMP activated protein kinase (AMPK) and mechanistic target of rapamycin complex 1 (mTORC1) that converge on autophagy initiating kinase complexes, such as the ULK1/2 complex and the class III PI3K complex [16]. In response to augmented AMP/ATP ratio, AMPK is activated, which in turn deactivates mTORC1 and/or provokes ULK1 and Beclin1 activity directly. The nutrient sensing kinase mTORC1 is a negative regulator of autophagy through inhibitory phosphorylation of ATG13 and ULK1 [16]. Monitoring of mTORC1 activity by checking the phosphorylation status of its substrates has revealed controversial results in EV infection. It was reported that infection with CVA16 and EV-A71 results in a reduction of mTORC1 activity [62,64], whereas CVB3 infection presented no evident changes in the activity of mTORC1 [60,76]. Consistent with the latter result, a recent study showed that phosphorylation and activity of mTORC1 do not change throughout PV infection [77]. Further study demonstrated that PV-induced autophagy is independent of the ULK1 complex [77], suggesting the existence of alternate mechanisms by which EV induces autophagy. Similarly, CVB3 was shown to benefit from a non-canonical form of autophagy that appears to bypass the requirement for core autophagy initiation components, such as Beclin 1, UVRAG, and ATG14 [68]. Finally, several studies revealed that viral components of the RNA replication machinery, such as viral proteins 2B, 2BC, 3A, and 3AB, are sufficient to induce LC3-lipidation and/or autophagosome formation [58,78–81]. Collectively, the current evidence suggests that EVs can employ multiple strategies to induce the accumulation of autophagosomes; however, the mechanism is likely independent of the canonical autophagy pathway.

## Fusion machinery of autophagy, EV maturation and release

As noted earlier, autophagy is a dynamic process starting with autophagosome formation, followed by fusion with lysosomes and consequent degradation of enclosed cargo. It is now clear that accumulation of autophagosomes could be due to increased induction of autophagosome biogenesis and/or reduced autophagosome fusion with lysosomes. To evaluate the effects of EV infection on autophagy flux (a measure of the completion of degradative autophagy), early research focused on monitoring the stability of the cargo receptor SQSTM1/p62 that is well known to be degraded during complete autophagy [82]. It was found that PV infection causes a decrease in protein levels of SQSTM1/p62, prompting the authors to conclude that PV infection enhances autophagy flux [83]. However, it was later revealed that SQSTM1/p62 is not only a substrate of autophagy, but also targeted by viral protease 2A during CVB3 infection [84], raising the concern whether it serves as a dependable marker to assess autophagy flux. The cleavage of SQSTM1/p62 was recently confirmed upon PV, EV-D68, and HRV1A infection [65].

Using different assays, including non-cleavable SQSTM1/p62, mRFP-GFP-LC3 tandem reporter, and electron microscopy, to evaluate flux, several groups have revealed that autophagosome-lysosome fusion is compromised during CVB3 and EV-D68 infection [59,85,86]. To address the underlying mechanism, the Jackson and Luo Laboratories worked in parallel to study the role of SNARE proteins in EV-D68 and CVB3 infection [65,85,87]. They found that SNAP29, a SNARE linker protein, is cleaved after amino acid 161 through the proteolytic activity of viral protease 3C. This cleavage dissociates the N-terminal STX17-interacting domain from the C-terminal VAMP8-binding motif, thereby disrupting the formation of STX17-SNAP29-VAMP8 complexes and consequent failure of autolysosome formation [65,85,87]. In addition to SNAP29, the Luo lab also discovered that (1) the protein levels of STX17 and VAMP8 are reduced late during CVB3 infection; and (2) the tethering adaptor protein PLEKHM1, another key component of the fusion machinery, is cleaved following CVB3 infection, all contributing to impaired autophagosome fusion [85]. Similarly, there was a report describing reduced autophagy flux after CVB3 infection as a result of decreased mRNA levels of STX17 [86]. Interestingly, in the study of EV-A71 infection, it was shown that viral protein 2BC physically binds to STX17 and such interaction was proposed to enhance autolysosome formation [88]. The reason for the discrepancy between EV-A71 and other major members of the EVs is not clear; but may be the result of differences in autophagy assays and possibly viral strains.

How do EVs benefit from disruption of autophagosome-lysosome fusion? It is plausible to assume that inhibition of a complete autophagy is a viral strategy to escape viral RNA/protein degradation. In addition, buildup of autophagosomes caused by an inhibition of autophagosome fusion and consequent degradation favors viral replication by offering replication membranes (see section above). It is also proposed that autophagosomes are re-directed to merge with late endosomes to form acidified amphisomes that are required for EV (e.g., PV, EV-D68, EV-A71) maturation to produce infectious viruses [65,83,88]. SNAP47 appears to play a role in autophagosome fusion with late endosomes in the absence of functional SNAP29 [65,87]. There is also evidence that EVs utilize autophagosomes and/or amphisomes to exit the infected cells [11,14,65,89].

EVs have traditionally been considered to escape the infected cell by causing it to rupture. However, emerging evidence highlights the significance of EVs in being able to spread through non-lytic mechanisms [90], such as via autophagosome-mediated exit without lysis (AWOL) [91]. Apart from its central role in cargo degradation, autophagy also has a function in unconventional secretion of cytosolic proteins, such as the release of inflammatory cytokines [92]. The ‘secretory autophagy’ is originally identified as an alternative disposal strategy under conditions of impaired lysosomal function [92]. A growing body of work has documented that EVs, including PV, EV-D68, EV-A71, CVB3, HRV2, can indeed hijack the autophagic machinery to usurp its abundant membranes by repurposing them as envelopes for non-lytic viral egress [11,12,14,65,89,93]. This strategy seems to provide unique advantages for EV [90]. For example, by cloaking inside host-derived vesicles, EVs adopt a Trojan horse-like strategy to propagate within their host organism, thereby shielding their pathogen-associated patterns from the adaptive immune detection. In addition, the use of quasi-envelopes that enclose multiple viral particles within a single vesicle was discovered to deliver viral particles more efficiently than infection with individual “naked” virions [12]. The mechanisms by which EVs use autophagy as a means of non-lytic spread is largely undefined. Recent studies on CVB3 and EV-D68 postulate that inhibition of autophagosome-lysosome fusion benefits viruses by re-directing autophagosome and/or amphisome vesicles from degradative autophagy to secretory autophagy, ultimately resulting in AWOL [65,85,87]. Similarly, there was a report describing that CVB3 infection induces the generation of mitochondrion-containing autophagosomes (coined mitophagosomes) [93]. Instead of being targeted and degraded by lysosomes, these mitophagosomes are released along with the enclosed viral particles from the infected cells [93]. However, the process of how autophagosomes and/or amphisomes are guided to merge with plasma membranes for viral release remains elusive. The SNARE proteins are known to have an important role in vesicle exocytosis via regulating membrane fusion [94]; however, the exact SNARE(s) that mediate docking and subsequent fusion of autophagosomes with the plasma membranes have not been identified, and warrants further investigation.

## Selective autophagy and EV evasion of host anti-viral immunity

It is well documented that autophagy are involved in both innate and adaptive immunity [37,95]. One of the best-appreciated functions for autophagy in immunity is to defend against microbial invasion [96,97]. For this reason, autophagy has traditionally been considered an anti-viral machinery. In particular, autophagy can selectively target invading viruses through a process, called virophagy, for clearance [16]. Similar to other types of selective autophagy, virophagy is mediated through autophagy receptors, including SQSTM1/p62, NBR1, optineurin, CALCOCO2/NDP52, and TAXBP1 [98]. The first-identified virophagy receptor is SQSTM1/p62 that was discovered to interact with capsid proteins of Sindbis and Chikungunya viruses, which are positive-sense RNA viruses in the *Togaviridae* family, and target viral particles and/or capsid proteins for autophagic degradation [99,100]. Recent studies on CVB3 have revealed that both SQSTM1/p62 and CALCOCO2/NDP52 are able to bind capsid protein VP1 to mediate virophagy [101]. Further characterization demonstrated that VP1 undergoes ubiquitination (a common signal for substrate recognition by autophagy receptors [102]) during infection, indicating a possible ubiquitin-dependent virophagy mechanism [101]. Although direct evidence is lacking, the early electron microscopy findings of PV and CVB3 virions inside the double-membraned vesicles suggest the possibility that SQSTM1/p62 and CALCOCO2/NDP52 can also recruit intact viral particles beyond capsid proteins to autophagosomes for degradation.

To counteract the anti-viral action of virophagy, EVs have developed strategies to directly target autophagy receptors. For example, infection with several EVs, including CVB3, EV-D68, PV, and HRV1A, has been shown to cause the cleavage of SQSTM1/p62 [65,84], suggesting a conserved EV strategy to subvert the host virophagy efforts. Existing literature also shows that CVB3 infection results in degradation or accumulation rather than cleavage of SQSTM1/p62 [59,68]. The discrepancy between studies is unclear [59,68,84]; it may be related to the differences in viral strains and cell types, as well as *in vitro* versus *in vivo* studies. Additional research demonstrated that NBR1, a functional homolog of SQSTM1/p62, is also cleaved upon CVB3 infection, excluding a possible compensatory role for NBR1 when SQSTM1/p62 is disturbed. Of note, it was found that cleavage of SQSTM1/p62 and NBR1 not only results in a loss-of-function but also produces a dominant-negative fragment against native proteins [103]. Recently, autophagy receptor CALCOCO2/NDP52 was identified as a novel substrate of CVB3 protease [101].

In addition to direct targeting of virus, autophagy receptors can also modulate immune signaling [36,37]. Study of CVB3 infection found that knockdown of SQSTM1/p62 results in enhanced viral growth, whereas gene-silencing of CALCOCO2/NDP52 leads to a reduction in viral yields, indicating an opposite role for these two proteins in CVB3 infection [101]. Further investigation has identified the pro-viral mechanism of CALCOCO2/NDP52, which inhibits the anti-viral type I interferon signaling by facilitating the degradation of the mitochondrial anti-viral signaling (MAVS) by autophagy [101]. Intriguingly, it was discovered that the cleavage fragment generated during infection retains the pro-viral function of full-length CALCOCO2/NDP52 [101].

Toll-like receptors (TLRs) are important mediators in innate anti-viral immunity. It is well characterized that EV infection activates the TLR signaling to initiate the host innate immune response [104]. Current evidence suggests a dual role for autophagy in the regulation of the TLR signaling during EV infection [105,106]. Research of CVB3 demonstrated that virus-activated, TLR3-dependent type I interferon signaling requires autophagy [105]. Results from this study support a model that autophagosomes fuse with TLR3-positive endosomes to form amphisomes, which serve as platforms to sense the viral pathogen-associated molecular patterns (delivered by autophagosomes) and consequently activate the downstream anti-viral signaling. In contrast, it was recently reported that enhanced autophagy in response to EV-A71 and CVA16 infection benefits viruses by inhibiting TLR7-mediated type I interferon signaling, although the detailed mechanism remains to be determined [106].

Collectively, current evidence highlights a convergence between EV subversion of the autophagy pathway and the host innate immunity that ultimately favors viral replication.

## Conclusion

EVs have strategically intertwined their life cycle with the host autophagy pathway in an effort to co-opt its abundant machinery for continuous propagation (see Figure 2). The limited RNA genome of EVs is sufficient to induce damage to various cell types across different tissues leading to broad human pathologies. With the exception of the successful vaccination program of PV across much of the globe, EVs continue to plague many nations, often targeting infants and children. As a result, large efforts are needed to develop novel and effective therapies against EV infection. The increased awareness that EVs hijack and disrupt an important cellular pathway such as autophagy is opening new opportunities for developing anti-viral therapy. Despite significant advances in our understanding of the EV-autophagy interplay, open questions remain that warrant further investigation. For example, what is the exact mechanism/upstream signaling by which EVs induce autophagosome biogenesis and membrane remodeling? What are the SNARE proteins responsible for the docking and/or fusion of enveloped EVs with plasma membrane? Is there a cross-talk between the host autophagy and endo-lysosomal pathway during EV infection and what is its role in viral entry and exocytosis? Uncovering these answers will provide insights into novel mechanisms of EV-host interaction, with the hope of leveraging future findings for more effective anti-viral therapy.
